# Readmission of Patients to Acute Psychiatric Hospitals: Influential Factors and Interventions to Reduce Psychiatric Readmission Rates

**DOI:** 10.3390/healthcare10091808

**Published:** 2022-09-19

**Authors:** Ernest Owusu, Folajinmi Oluwasina, Nnamdi Nkire, Mobolaji A. Lawal, Vincent I. O. Agyapong

**Affiliations:** 1 Department of Psychiatry, University of Alberta, Edmonton, AB T6W 3W8, Canada; 2 Department of Psychiatry, Dalhousie University, Halifax, NS B3H 4R2, Canada

**Keywords:** readmission, psychiatry, patient, acute hospital, intervention

## Abstract

**Background:** Appropriate and adequate treatment of psychiatric conditions in the community or at first presentation to the hospital may prevent rehospitalization. Information about hospital readmission factors may help to reduce readmission rates. This scoping review sought to examine the readmission of patients to acute psychiatric hospitals to determine predictors and interventions to reduce psychiatric readmission rates. **Method:** A scoping review was conducted in eleven bibliographic databases to identify the relevant peer-reviewed studies. Two reviewers independently assessed full-text articles, and a screening process was undertaken to identify studies for inclusion in the review. PRISMA checklist was adopted, and with the Covidence software, 75 articles were eligible for review. Data extraction was conducted, collated, summarized, and findings reported. **Result:** 75 articles were analyzed. The review shows that learning disabilities, developmental delays, alcohol, drug, and substance abuse, were crucial factors that increased the risk of readmission. Greater access to mental health services in residential treatment and improved crisis intervention in congregate care settings were indicated as factors that reduce the risk of readmission. **Conclusion:** High rates of readmission may adversely impact healthcare spending. This study suggests a need for focused health policies to address readmission factors and improve community-based care.

## 1. Introduction

Over the past fifty years, ‘deinstitutionalization’ has led to a more significant and faster transition from inpatient psychiatric care to treatment in the community [1]. Notwithstanding the success story of deinstitutionalization, many patients have had severe problems after being discharged from psychiatric hospitals, which increased the number of psychiatric emergency readmissions to the acute wards [1]. The increase in readmission has, over the years, put a lot of pressure on the few psychiatric facilities and the limited resources at their disposal [2], and posed a significant challenge in psychiatry for the last decades [3,4]. Psychiatric readmission reduces patients’ quality of life and increases the years of life lost in care [5].

A better understanding of the factors related to readmission may help to improve the management of patients in inpatient treatment facilities and also put in measures to intervene early to reduce readmission rates and thereby save cost [6]. Most patients seeking rehospitalization tend to repeat some of these treatment-seeking behaviors and may assist nurses in planning care [7,8].

It is estimated that about one-third of patients admitted to psychiatric services will probably be readmitted within a year [6]. As shown in previous studies, psychiatric readmission rates differ by country and different age groups. For example, the rate is 22% in America for psychiatric patients aged over 65 years [5] and 19% for children admitted to the emergency ward in Baltimore aged 4–18 years [9]. The rate is 14% of patients admitted to the psychological ward of the army in Washington [10], 33.7% of patients diagnosed with schizophrenia in Turkey [11], and 43% of teenage patients admitted for psychological reasons in Northern Carolina [4]. The readmission rate may be used to measure the extent of the quality of care rendered to patients which is also the focus of interest for all health sector policymakers [12,13]. The readmission rate is also used as the determinant of quality of care for measuring outcomes such as continuity of care and follow-up services.

The concept of the readmission period has been defined differently in different studies. For example, readmission within 90 days [14], readmission within 3 years [10], admission three or more times within 30 months [15], readmission within 6 months [16], and admittance of 3 or more times within 2.5 years [7]. This has resulted in a variance in the reported rates of readmission in different studies as 14%, 16% [15], 20–30% [10], and 45–53% [16]. Demographic, social, and clinical factors and their effects on possible readmission have been studied for more than thirty years [17]. Poor treatment adherence [18,19], non-voluntary first admission [7,18], and substance, alcohol, and drug abuse [4] have been identified as related factors to the readmission of patients to psychiatric wards. Other demographic characteristics such as marital status, unemployment [20,21], retirement [20], and gender [19] are also considered relevant factors that can influence readmission.

Some studies have illustrated that specific diagnoses may be associated with readmission. For example, the diagnosis of schizophrenia has been reported as a significant factor in readmission. The diagnosis of bipolar disorder [4,16], psychotic disorders [9,22], and mood disorders [3], also influence readmission. In some studies, diagnoses with more psychiatric comorbidities [6] were also a factor associated with readmission. Suicidal ideation or thoughts of self-harm [6], history of psychological problems in childhood, and the first episode of the psychiatric disorder under 18 years have been revealed as related factors for readmission [9]. The length of stay in the hospital during admission has also been observed to impact readmission [7,18]. The shorter the length of the stay at a hospital- perhaps due to the lack of empty beds, the more likely the readmission of psychiatric patients. This can affect the quality of care and increase the costs of service to the provider [12]. In other studies, the history of admission in elderly patients has been identified as influencing readmission [6,9].

The role of the disease course and severity and its correlation to readmission also remains unclear, with various findings regarding primary diagnosis and symptom severity [22], comorbidity [23], self-injury, and suicidality [5].

The characteristics of the hospitalization may also predict readmission, possibly the length of stay [6,16] (LOS), having access to inpatient case management services [21], and aftercare services upon discharge from the hospital, including home care and home visit services [18], disposition to residential treatment or partial hospitalization [21], and discharge medications [22,23].

Studying the influential factors relating to readmission may help to improve management interventions. Identifying predictors of readmission at this level can inform health policies and quality improvement interventions to mitigate cost and the burden to systems and families [23].

This scoping review seeks to elucidate variables that impact patients’ readmission into acute psychiatric hospitals and propose interventions to reduce readmission rates.

## 2. Methods

This review was planned and carried out in adherence to the Preferred Reporting Items for Systematic Reviews and Meta-Analyses Extension for Scoping Reviews (PRISMA-ScR) statement [24]. This study adopted a comprehensive search strategy that allows for reproducibility, reliability, and transparency in the current state of literature. The review was conducted in five stages, described below:

### 2.1. Stage 1: Developing the Research Question

Our research question was, “What is known about the readmission of patients in psychiatric hospitals and what are the influential factors and interventions to reduce the psychiatric readmission rate?”

### 2.2. Stage 2: Identifying the Relevant Studies

We used the PRISMA extension for scoping reviews to identify the included studies. The checklist contains 20 essential reporting items and two optional items to include when completing a scoping review. Original peer-reviewed articles from databases’ inception until December 2021 were obtained from systematic searches of several electronic bibliographic databases, including Medline via OVID (1946–Present), EMBASE via OVID (1974–Present), PsycINFO via OVID (1806–Present), Global Health via OVID (1910–Present), HealthSTAR via OVID (1966–Present), Cumulative Index for Nursing and Allied Health Literature (CINAHL) via EBSCOhost (1936–Present), Environment Complete via EBSCOhost (1950–Present); Scopus via Elsevier (1976–Present), Sociological Abstracts via ProQuest (1952–Present), Dissertations and Theses Global via ProQuest (1861–Present), and Cochrane Library via Wiley (1993–Present). Both natural language terms and controlled terms were derived from three main concepts: (1) Readmission of patients into acute psychiatric hospitals; (2) Influential factors for readmission rates; and (3) Interventions to reduce psychiatric readmission.

**Figure 1:**
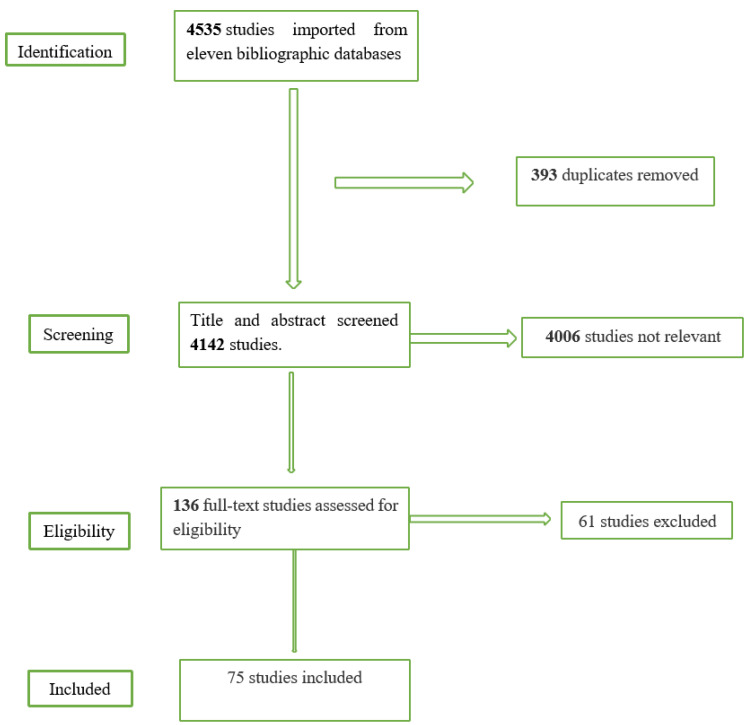
PRISMA flow chart.

In total, 4535 records were identified through database searches. Duplicate records were automatically removed upon import to the systematic review management software, Covidence. In total, 393 duplicate records were identified. The complete search strategy has been attached as an appendix (Figure 1).

### 2.3. Stage 3: Article Selection

Based on eligibility criteria, the first selection was the screening of the title and abstract and the second screening was a full-text review. A third reviewer resolved all conflicts generated through the screening stages between the reviewers. Each eligible article met the following inclusion criteria: (1) focus on the readmission of patients in psychiatric hospitals; (2) Discusses predictors or influential factors for readmission; and (3) Discusses interventions to reduce psychiatric readmission rates.

Grey literature and review articles that were not peer-reviewed were excluded. No language or publication restrictions were applied. We identified 136 articles for full-text review. The full-text review generated 75 articles. Upon reviewing the 136 full-text articles, sixty-one (61) articles were excluded as they did not meet the inclusion criteria. The PRISMA flow diagram summarizes this information in detail (Figure 1).

### 2.4. Stage 4: Data Charting and Data Extraction

The following information was extracted from 75 articles: author(s) name, year of publication, country of study, study type, sample size, age, type of psychiatric condition, finding, and conclusion.

### 2.5. Stage 5: Collating, Summarizing, and Reporting the Results

We present an overview of the existing evidence relating to the readmission of patients in acute psychiatric hospitals and review the available literature on the influential factors and interventions to reduce such readmission rates. This review has been reported according to the Preferred Reporting Items for Systematic Reviews and Meta-Analyses (PRISMA), extension for scoping review guidelines. The characteristics and results reported in each included article were summarily described.

## 3. Result

### 3.1. Characteristics of the Studies Included

In all, seventy-five (75) articles were included in this review.

The characteristics of the articles are presented in Table 1. The majority, 68% (51), were cross-sectional studies (retrospective and descriptive), 21.3% (16) were qualitative studies, 2.7% (2) were mixed methods, 4% (3) were randomized clinical trials, and 4% (3) of the research methods were not specified. The study population size in the included articles ranged from 5 to 400 participants. Study participants’ age ranged from 8 years to >75 years as reported in the studies.

**Figure 2:**
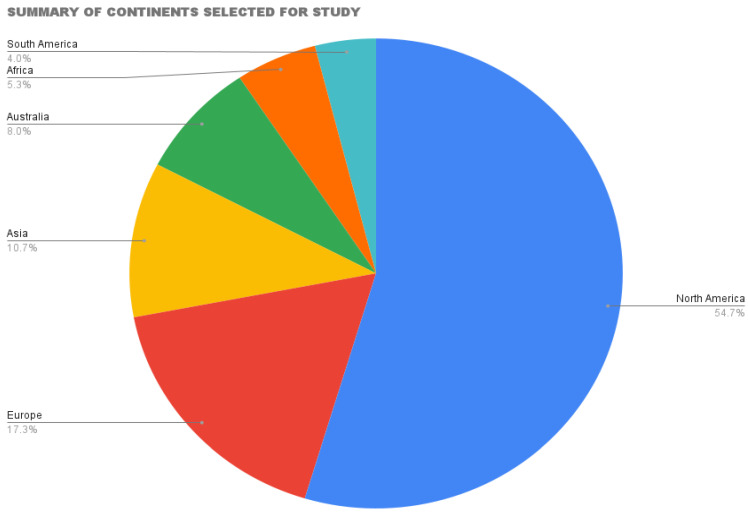
Summary of Continents selected for Study.

This scoping review included studies from 1946 to 2021. The majority (82%) of the studies included were published in the last twenty-one years, mainly in English. Slightly more than fifty percent of the studies were conducted in North America (55%), Europe 17%, Asia 11%, Australia 8%, Africa 5%, and the remaining 4% were from South America. (Figure 2). The majority (82%) of the studies examined acute psychiatric conditions, while 18% of the studies were on general psychiatric conditions. 

### 3.2. Influential Factors for the Readmission of Psychiatric Patients

#### 3.2.1. Length of Hospitalization for First Admitted Psychiatric Patients

Six studies (6) [25,26] reported that hospitalization characterized with short periods of inpatient stay, especially for first admitted patients, were associated with higher risk of readmission [27]. The short length of stays can be helpful in reducing costs in managing patients in the early stages of mental disorders [26,27] when there is an effective integration between inpatient units and community-based services [26]. The risk of readmission was higher in the periods immediately after discharge [28]; the further away from the discharge, the lower the risk of a new admission [25]. In a study [29] conducted and published by Tedeschi et al., 2019, the predictors for readmission were a shorter length of stay (LoS); the protective factors identified in the study were young age, involuntary admission, and the intermediate number of public healthcare staff available (28). The findings also highlighted that individual-level factors mainly determined readmission [28,30]. 

#### 3.2.2. Patients with a Previous Diagnosis of Chronic Psychiatric Disorders

Twenty-one studies (21) reported that the significant predictors of early readmissions were a clinical diagnosis of depressive disorder, bipolar disorder, schizoaffective disorder [31], psychotic disorder, seasonal affective disorder (SAD) [32], and non-alcohol-related disorder [33]. The demographic features such as younger ages and unemployment [34,35] which are perceived to be related to the severity of the mental disorder were also identified as predictors for readmission [25,35]. A study by Smith et al., 2015 reported that a high rate of readmissions was significantly associated with certain conditions on the index visit, namely, alcohol or drug dependence, dementia, psychotic disorders, autism, impulse control disorders, and personality disorders [31,36].

Downey et al., 2015 reported that most patients readmitted to the hospital through the emergency department within 90 days were readmitted for the same psychiatric illness [35]. A study [22] found that the majority (90%) of patients who were readmitted to the psychiatric ward presented within the top five diagnoses of depression, schizophrenia, schizoaffective disorder, bipolar disorder, and psychosis [37].

In a cross-sectional study conducted by Manu et al., 2014, a quarter of the subjects had a relapse requiring readmission within one year of admission following treatment for a severe mental illness [38]. The study findings also showed an independent predictor correlated with high readmission rates, including: a higher body mass index (BMI), a diagnosis of schizophrenia or schizoaffective disorder, treatment with clozapine, shorter length of stay, and no electroconvulsive (ECT) therapy during the index admission (37). The independent predictor for psychiatric rehospitalization identified by the study was the body mass index (BMI) (37). Effective outpatient treatment for psychiatric patients with problems with weight and obesity might help influence readmission rates downwards and should be explored in prospective studies. Lorine et al., 2015 in their study indicated that a diagnosis of schizophrenia/schizoaffective disorder, history of increased alcohol use, the number of previous psychiatric hospitalizations [39], and the type of residence at initial admission (e.g., homelessness) were found to be significant risk factors for early readmission [40]. 

#### 3.2.3. Presence of Learning Disability, Developmental Delay, and Personality Disorder

In a study conducted by Romansky et al., 2003, it was established that the only clinical variable that delayed the risk of readmission between the children who were readmitted and those who were not was the diagnosis of a learning disability or developmental delay [41]. Children with mild mental retardation were more likely to be admitted than those who were not [41]. This study established a significant association between enabling characteristics, mainly environmental and service delivery factors, and the readmission of children and adolescents in the child welfare system [41]. 

A previous psychiatric hospital admission was associated with an increased readmission rate among people diagnosed with a personality disorder and was associated with the highest rate of readmission [42]. The study suggests that other factors predicting readmission were an increased deprivation, taking medicines for chronic disease, drug dependency, or antidepressants. In a quantitative study [43] that involved 2894 people presenting to the hospital, patients with personality disorders represented 20.5% of emergency cases and 26.6% of the total inpatients. Patients with a personality disorder or psychosis were 2.3 times more likely than others to represent within 28 days [44]. A personality disorder diagnosis increases the readmission rate by a factor of 8.7, marginally lower than psychotic disorders [43]. Another study [43] reported that personality disorders place significant demands on inpatient and emergency departments, similar to psychoses in terms of presentation and risk of readmission. Personality disorders carried the most significant risk of readmission among psychiatric patients [36].

### 3.3. Alcohol, Drugs, and Substance Abuse

Thirteen studies (13) reported that the influential factors for readmission were due to an alcohol-related psychotic disorder [27,45]. This condition is caused by chronic alcohol abuse, followed by abrupt alcohol cessation. Although the condition is rare [34], it’s characterized by auditory, visual, or tactile hallucinations paired with intact orientation and stable vital signs. These distinguishing factors separate the condition from other psychotic disorders or delirium tremens (DTs). In a study conducted by Soyka et al., 2013, the number of patients treated for an alcohol-induced psychiatric disorder (AIPD) ranged from 390 to 486 annually (2005–2010) [42]. The number of cases recorded per year is more than double this figure, indicating a significant risk of relapse or chronicity in mental or behavioral disorders, which has caused high hospital readmission rates. Other studies [36,45] reported that other reasons for the high number of readmissions might be generally the high comorbidity in patients with AIPD, as demonstrated by Soyka et al. who reported that the alcohol relapse rate is increased since AIPD is the consequence of chronic and severe alcohol dependence [42]. Results from this study [46] question these earlier findings and indicate a strikingly high rehospitalization rate for patients with AIPD.

In conclusion, the data from these hospital statistics [46] indicate that AIPD is a relatively ‘‘stable’’ clinical syndrome with a high rehospitalization rate and a more chronic relapsing course than previously thought. Although it cannot be concluded that AIPD is the only cause for readmissions as AIPD is the consequence of chronic and severe alcohol dependence with an often high comorbidity. Nevertheless, patients should be more closely monitored during follow-up [42]. 

A younger age, higher deprivation, the use of certain drug groups or multiple drug types, and prior psychiatric hospital admission are all well known risk factors for readmission with self-poisoning [2]. In a study conducted by Mellesdal et al., 2010, substance use disorder [47] was a significant predictor for readmission. Four studies [48,49] included in this scoping review suggest that psychiatric hospitalization and readmission are frequent among drug-addicted patients [50,51]. 

A qualitative study by Moos et al., 1998 exploring the rates and predictors of four-year readmission amongst late middle age, substance abuse patients, showed that four-year readmission rates in three diagnostic subgroups (alcohol or drug dependence diagnosis only, alcohol or drug psychosis, substance dependence and/or psychoses with one or more psychiatric diagnoses) were very high, ranging from 57 to 70% [52]. However, they were somewhat lower among patients with more minor chronic substance abuse problems. Readmission and multiple readmission were associated with a younger age, unmarried status, frequent prior service use, alcohol psychosis or psychiatric diagnoses, treatment in a psychiatric unit, and shorter hospital stay [49].

Rehospitalization for inpatient substance abuse or psychiatric care can result in substantial health care costs [42]. However, little is known about readmission and multiple readmission rates among older substance abuse patients; and how these rates differ among different diagnostic subgroups. Whereas almost 60% of the alcohol- or drug-dependent patients received specialized care in substance abuse units, only 43% of the patients with alcohol or drug psychoses and only 24% of the patients with psychiatric disorders [51]. Substance abuse patients with psychiatric diagnoses were more likely to receive specialized care in a psychiatric unit. 

According to a study conducted by Moos et al.,1994, out of sixty patients with only alcohol- or drug-dependence diagnoses, 25% were seen for seven days or less, and 31% were seen for 29 days or more. However, of the patients who also had psychiatric disorders, only 16% were seen for seven days or less, and 40% were seen for 29 days or more. These relatively long episodes of care occurred because the inpatient facilities were located in full-service medical centers. The same episode of care treatment was provided for medical complications for substance abuse, associated medical disorders, and medical rehabilitation [51]. Concerning the type of discharge, about 11% of the patients in each group were discharged against medical advice. Patients with more severe disorders were less likely to leave treatment against medical advice. The number of inpatient treatment episodes in the year before the index episode was the strongest predictor for readmission [51]. Unmarried, substance abuse patients were more likely to be readmitted [49].

However, in the context of the other predictors, these variables were not independently associated with readmission. Patients who had an alcohol or drug psychosis or a psychiatric diagnosis in the index episode were more likely to be readmitted [49].

### 3.4. Non-Medication Adherence

Eight studies (8) reported that non-medication adherence [53] was a predisposing factor for readmission of psychiatric patients [18]. Intentional medication non-adherence at home and diagnosis of epileptic psychosis demonstrated associations with readmission in a study conducted by Barnett et al. [54]. However, upon completion of a multivariate logistic regression analysis, the report of intentional medication non-adherence at home was associated with readmission. In a study [46] conducted by Brian et al., 2019, the findings suggested an association between intentional medication non-adherence at home and readmission during the study period [6]. 

Intentional medication non-adherence is common among psychiatric patients, especially those with schizophrenia [47]. However, the dataset used did not allow us to determine the reasons for intentional non-adherence among psychiatric patients. They were able to differentiate intentional non-adherence from unintentional non-adherence, such as when patients were taking their medications but ran out of them and were unable to obtain refills [45].

### 3.5. Suicidal Ideation

Ten studies [10] reported suicidal ideation as one of the major reasons for readmission rates [11]. The studies identified three important predictors associated with adolescent psychiatric rehospitalization: a diagnosis of PTSD, the severity of lifetime suicidal ideation (SI) [27], and a weak treatment alliance before hospitalization. Important areas for further research in adolescents in psychiatric crisis should include examining the role of PTSD, the influence, severity, and trajectory of self-injurious thoughts and behaviors (SITBs), especially SI, and the relationship of adolescent patients with their outpatient mental health providers [30]. Additionally, a prospective examination of the critical post-discharge period in order to better understand the predictors of negative outcomes in this population is warranted and should include longitudinally evaluating patients throughout treatment episodes that span multiple levels of psychiatric care [29].

Xu et al., 2018 reported an elevated risk of readmission for suicide in patients with epilepsy that is almost five times greater than for patients recently hospitalized for a stroke and three times greater than for patients hospitalized for medical comorbidities at index admission [53].

The findings further showed that admissions for epilepsy might be independently associated with more than a threefold increased risk of hospital readmission for suicide in the year following the index admission compared with patients recently hospitalized for stroke or other common medical disorders. Interestingly, epilepsy remained a strong predictor of suicide readmissions even after the adjustment for psychiatric history [54].

In a prospective cross-sectional study conducted by Czyz et al., 2016, investigating the course of suicidal ideation and future suicide attempts, it was revealed that rehospitalization was associated with a greater risk of suicide attempts [55]. Furthermore, readmission predicted distinct changes in suicidal ideation trajectories [25] within the elevated–fast declining and chronically elevated groups, and rehospitalization predicted increases in suicidal ideation during the follow-up, with a larger magnitude for the chronic group [29]. In contrast, rehospitalization was associated with a decrease in follow-up suicidal ideation in a subclinical group [30], while rehospitalization predicted a more severe course of suicide ideation for most adolescents [33]. However, it was protective for only a smaller subgroup with subclinical levels of suicidal ideation at index hospitalization [30]. A study [26] also suggests that rehospitalization is a strong indicator of the future risk of a suicide attempt.

Mellesdal et al., 2010 indicated that 54% of index admissions and 62% of the readmissions were related to suicide risk [47]. Personality disorders, prior psychiatric hospitalization, unemployment, and receipt of social benefits were significant predictors for readmissions because of suicide risk. At index admission, suicidal ideation or suicide plans significantly predicted readmission because of suicide risk. A higher number of readmissions per individual patient was associated with a greater tendency of admission because of suicide risk [37].

Their findings highlighted the importance of the targeted screening of patients with epilepsy for suicide risk at discharge from the index admission, even in patients without a preexisting psychiatric history [17].

### 3.6. Interventions to Reduce Psychiatric Patients’ Readmission Rates

#### 3.6.1. Residential Treatment Services

Romansky et al., 2003 reported that a greater access to sophisticated mental health services for children in residential treatment would reduce the risk of readmission, and an improved crisis intervention in congregate care settings should reduce the risk of readmission [20,56]. A residential treatment, a live-in health care facility providing therapy for substance use disorders, mental illness, or other behavioral problems, would help reduce the readmission of psychiatric patients [42,57]. Residential treatment may be considered the “last-ditch” approach to treating an abnormal psychology or psychopathology [42,58].

#### 3.6.2. Rendering Sufficient Inpatient Care

In a study conducted by Williams et al., 2015 [59], the findings suggested that rendering adequate inpatient care by addressing the acute presenting problem and stabilizing the patient’s psychiatric status [59,60], is an excellent intervention to address the readmission rate [61,62].

#### 3.6.3. Adequate Discharge Plan

In a qualitative study that examined the strategies to reduce psychiatric readmissions, the finding revealed that having an adequate discharge plan [63,64] and delivery of sufficient support services to transition psychiatric care successfully from an inpatient to an outpatient setting (e.g., discharge services, follow-up calls, short-term case management, bridge visits, and psychoeducation) has the potential to reduce readmission [65,66].

#### 3.6.4. Strengthen Focus on Staff Training

In a study conducted by Mellesdal et al., 2010, care planners and clinicians probably need to strengthen their focus on staff training [67] and supervision for assessing and managing suicidal patients and for collaboration with referring physicians and aftercare providers [47].

#### 3.6.5. Focus on Care Coordination and Transitional Efforts

To reduce these rates, initiatives should focus on care coordination and transitional efforts to gain appropriate outpatient care for those with these specific mental health and substance abuse (MHSA) conditions [68]. Enhanced care for outpatient treatment, especially for overweight and obese psychiatric patients, might influence readmission rates and should be explored in prospective studies [38].

#### 3.6.6. Promoting Psychological Support

In a study by Lewis et el., 1990, in addition to the number of previous readmissions, informal help for psychological distress was associated with readmission [36]. This study indicates that patients’ perceived needs could be considered to improve specific and individualized support and thus prevent a relapse and readmission. Moreover, it suggests that interventions in patients at risk of readmission, that focus on need assessment and promoting psychological support of the informal network, could effectively enhance the quality of life of patients with severe mental disorders [69].

#### 3.6.7. Adherence to Medication

Adherence to medication can be explained as the extent to which a patient’s behavior corresponds with the prescribed medication dosing regimen. It also determines the patient’s commitment to use the medication as prescribed by the physician. It includes the elements the time, dosing, and interval of medication intake [70]. Non-adherence is a crucial determinant for the success and otherwise of many therapies [71,72].

The commitment of staff and patients to improve medication adherence among patients following hospital discharge may help to decrease the possibility of readmission [52].

## 4. Discussion

This scoping review identified a wide range of studies examining the influential factors and interventions to reduce readmission of patients into acute psychiatric hospitals. The analyzed variables were classified according to the following categories: influential factors and the interventions to reduce these readmission rates. The following were identified as influential critical factors to the readmission of patients into acute psychiatric hospitals: a short period of hospitalization for first admitted patients, patients with previous chronic psychiatric disorders, alcohol, drug, and substance abuse, the presence of disabilities, developmental delay and personality disorders, non-adherence to medication and patients with suicidal ideation.

The length of stay, was analyzed as a predictor in some of the papers reviewed; the association of the LoS with readmission was significant and turned out to be a protective factor for first-time readmissions among patients [73,74]. Furthermore, other relevant aspects of the discharge process, such as adequate discharge plans and well-coordinated and focused transitional efforts, are essential in reducing the rate of readmission of patients into acute psychiatric hospitals [75,76].

Patients with previous chronic psychiatric disorders have been found to predict the likelihood of readmission into acute psychiatric hospitals [77]. Adequate and effective inpatient treatment and well-coordinated psychological care and medication management after discharge are crucial to reducing the risk of readmission [78].

The majority of patients readmitted were patients with suicidal ideation or plans, and this subgroup also had an increased risk of readmissions as a result of the risk of self-harm. For some patients in the suicidal ideation or plans group, the risk of suicide may be low, although their need for help may be substantial. Others in this subgroup may be at high risk of suicide. This study identified a small subgroup of patients who had a high number of readmissions because of the suicide risk [7]. Other studies have found the same pattern [79]. To some extent, this pattern may indicate that these patients experience suicidal behavior to communicate and regulate feelings and as a way of getting help when more constructive coping strategies are insufficient [7]. The findings indicate that a higher number of readmitted patients were related to an increased tendency to present with suicide risk [80]. Suicide attempts and non-suicidal self-injurious behavior increased among the most frequently readmitted patients. Clinical implications postulated by these findings are that clinicians as well as practitioners should focus on ascertaining whether suicidal ideation or planning indeed indicates a genuine death wish, or a pattern of attention-seeking behavior. Regardless, suicidal behavior is always a cry for help and should be taken seriously by clinicians, and careful exploration of the help being sought may provide important information in planning the treatment of such patients concerning implementing protective measures and psychosocial and psychopharmacological interventions. This will eventually help reduce the rate of readmissions involving such patients into acute psychiatric hospitals.

An association between non-adherence to antipsychotic medication shortly after discharge and early rehospitalization has been established, and several factors could be attributed to this phenomenon [80]. A study also suggested that the short duration of the first hospitalization for schizophrenia or schizoaffective disorder and the non-adherence to antipsychotic pharmacotherapy early after discharge, is associated with a higher risk of rehospitalization [80]. The lack of education on medication adherence and the lack of psychosocial support could be two of several reasons discharged patients may not adhere to their medications. Putting in place an adequate discharge plan [39] and delivery of sufficient support services to transition psychiatric care successfully from an inpatient to an outpatient setting can reduce the risk of readmission. For example, discharge follow-up services, follow-up calls, bridge visits, and psychoeducation have been identified to reduce readmission risk [62].

Some studies found very high 4-year readmission rates: 57% for those with only an alcohol dependence diagnosis, 64% for those with alcohol psychosis, and 70% for those who had a comorbid psychiatric disorder in the index episode [81]. These rates are considerably higher than those identified in the follow-ups of generally comparable groups of mixed-age and younger patients [82,83]. Some other studies found that lower readmission rates (41, 50, and 52%, respectively) among new substance abuse patients (those who had no inpatient substance abuse or psychiatric episodes in the four years before their index episode) [51,82]. These findings support the idea that older substance abuse patients with a more recent onset of substance abuse problems may have a better prognosis than those with more chronic difficulties [51]. In addition, they underscore the importance of distinguishing late-onset and early-onset substance abuse in evaluations of treatment programs for older patients [82]. Rendering sufficient inpatient care by adequately addressing the acute presenting problem and stabilizing the patient’s psychiatric status was an excellent intervention to address the readmission rate, most especially for alcohol and substance abuse patients. Furthermore, promoting psychological and a good support network for patients discharged for substance abuse can help reduce the risk of readmission. Moreover, the interventions for patients at risk of readmission that focus on need assessment and promoting psychological support of the informal network could be effective in enhancing the quality of life of patients with severe mental disorders, including substance use and alcohol-related psychotic disorders [41].

## 5. Limitations

This review, like any other review of this nature, has some limitations. A limitation to this scoping review is that the authors did not document or register the protocol for the review and so it is not published as part of this review in order to allow for reproducibility. Another limitation was that many articles discussed the concept of readmission without identifying influential factors or interventions to reduce readmission. Moreover, our search strategy considered articles written in English alone which means other articles written in another languages with valuable information might be lost.

## 6. Conclusions, Implications for Policy and Practice

In conclusion, the factors identified to influence the readmission of patients into acute psychiatric hospitals cannot be overemphasized. The implementing strategies to mitigate these factors will reduce the rate of readmission of patients into these acute psychiatric facilities. In line with factors identified in this review, it is recommended that residential treatment, provision of sufficient inpatient care, and adequate discharge planning are necessary prerequisites prior to the discharge of patients. Furthermore, it is recommended that strengthened in-service training in order to upskill staff on new and contemporary knowledge-based practices and strict medication adherence monitoring should be implemented to reduce readmission rates. In addition, the focused and coordinated transitional care programs can be implemented as part of discharge plans. For example, use of peer support services which have been found to be effective in reducing readmission rates [83,84] can be implemented as part of a discharge planning program to promote psychological support services for discharged patients at home. Governments, policy-makers, and health authorities can also adopt and implement population-level interventions such as the use of supportive message programs such as Text4Hope [85], Text4Mood [86] and Text4Support [87] which have been found to be effective in providing psychological support services [88] for the general public and patients in the community. In two randomized controlled trials, patients with a major depressive disorder who received twice daily supportive text messages, had significantly lower depressive symptoms compared with usual care patients [89]. Furthermore, the Text4Hope program, implemented in Alberta during the COVID-19 pandemic, was found to be effective in reducing symptoms of stress, anxiety, depression, and suicidal ideation in subscribers [90,91,92]. Supportive text messaging programs are evidence-based, cost-effective, easily scalable, devoid of geographical barriers to access, do not require expensive data plans, and comes at no cost to subscribers. There is currently a large trial in Edmonton to investigate the impact of supportive text messages, with or without peer support, on readmission rates for patients discharged from acute psychiatric hospitals in Alberta [93]. The outcome of this study will provide insights into the potential for using these low cost and easily scalable interventions to reduce readmission rates for patients discharged from inpatient psychiatric units.

## Figures and Tables

**Table 1:** Characteristics of included studies.

Authors	Country	Study Type	Sample Size	Age Range	Medical Conditions	Study Findings
Albrecht et al., 2012	USA	Retrospective cohort study	26,878	58 years	Severe mental illness/diabetes	No significant association between Serious mental illness (SMI) and 30-day hospital readmission was found in any group.
Bach et al., 2002	USA	Retrospective comparative study	35	39.2 years	Schizophrenia, depression, and bipolar disorder	Four sessions of an individual acceptance intervention reduced the rate of hospitalization over four months by 50% in a chronic group of hospitalized patients experiencing positive symptoms of psychosis.
Bardach et al., 2020	USA	Chart review	22,844	6–17 years	Depression, bipolar disorder, psychosis, and anxiety	The readmission rates were 62% for 7-day, and 82.3% for 30-days. Acute care readmission rates were 22.4% within 30 days and 54.8% within six months.
Barker et al., 2018	Canada	Retrospective chart review	65,789	46 years	Psychiatric diagnosis	The 30-day readmission rates were 9.3% and 9.1% for women and men, respectively. Predictors were personality disorders and positive symptoms for women and self-care problems at admission and discharge and mild anxiety for men.
Barnett et al., 2020	Malawi	Retrospective chart review	419	10–74 years	All patients on admission	Ten percent were readmitted at least once during the study period. The predictor was the intentional medication non-adherence at home.
Batalla et al., 2013	Spain	Retrospective chart review	58	18–29	Substance use disorder	Cocaine and cannabis use disorders were predictors for readmission.
Behr et al., 2002	South Africa	Retrospective cohort study (analytical and descriptive)	180	21–50 years	All psychiatric diagnoses	The rate of readmission within the 90 days post-discharge was 21%. Being married or co-habiting was found to be a protective factor for readmission.
Boag et al., 2021	USA	Retrospective cohort design	5076	45.2 years	Psychiatric diagnosis	The 180 days readmission rate was 23%. Prediction models incorporating topics derived from Latent Dirichlet Allocation in addition to simpler bag-of-words features performed comparably to, or better than, the models relying solely on bag-of-words or coded data.
Botha et al., 2018	South Africa	Randomized, non-blinded clinical trial	100	18–59 years	Schizoaffective disorder or bipolar disorder	The rate of readmissions over the 12 months was 34%. Participants in the facilitated care group (FCG) appeared to be more likely to have more than one readmission (18%) compared to the treatment as usual group (TUG) participants (7%).
Callaly et al., 2010	Australia	Retrospective cross-sectional study	115	Adult	All psychiatric conditions	Predictors for readmission were being admitted in the previous year, receiving the Disability Support Pension, not having a discharge plan and being unemployed. The rates for readmission were 45% within seven days, 68% within 14 days and 91% within 21 days.
Carlisle et al., 2012	Canada	Population-based cohort analysis	4472	16.9 years	Psychiatric diagnosis	No aftercare in the month post-discharge increased the risk of readmission but not emergency department visits.
Cheng et al., 2017	Canada	Retrospective analysis	15,628	5–24 years	Psychiatric conditions	Fourteen percent (14%) were readmitted within 90 days. Readmission predictions were prior service use, more extended hospital stays, higher income, specific diagnoses, female sex, and comorbid mental health conditions. Aftercare reduced the risk of readmission by 32%.
Conley et al., 1999	USA	Retrospective cross-sectional study	2024	41.6 years	Schizophrenia and other mental conditions	The predictor for readmission was schizophrenia. The readmission rate for risperidone-treated patients appeared to be steady up to 24 months. At 24 months, 87% of the clozapine-treated patients and 66% of the risperidone-treated patients remained in the community.
Czyz et al., 2016	USA	Prospective cross-sectional study	373	13–17 years	Substance use disorder with risk of suicide	The 90 days readmission rate was 21%. Youths with a history of multiple suicide attempts were the predictors.
Di Giovanni et al., 2020	Italy	Retrospective observational study	325	36.5 years	Drug addiction	Overall, 80.8% of readmissions occurred during the first year and 88.2% during the first two years. The critical predictor was drug-addicted patients.
Doering et al., 1998	Germany	Retrospective chart review	354	18–55 years	Schizophrenia and schizoaffective disorder	Readmission predictors were neuroleptic treatment, onset and previous course, psychopathology, social adjustment (marital status, employment, intensity of life), previous life experiences and biology (gender, age).
Downey et al., 2015	USA	Retrospective chart review	350	30–49 years	Depression, schizophrenia, schizoaffective disorder, bipolar disorder, and psychosis	The predictors for readmission were depression, schizophrenia, schizoaffective disorder, bipolar disorder, and psychosis.
Guzman-Parra et al., 2018	USA	Longitudinal observational study	780	18–78 years	Psychiatric conditions	The rate of readmission within one year was 36.28%. The overall rates of readmissions within 30, 90 days, and one year after discharge were 21.21, 40.40, and 61.61%, respectively. Predictors were previous readmissions and informal help for psychological distress.
Haddad et al., 2020	Lebanon	Retrospective observational study	158	43.7 years	Acute mania	Predictors for readmission were male gender, taking anticholinergic drugs, and having a family history of psychiatric disorders.
Hamilton et al., 2015	USA	Retrospective chart review	588	18–71 years	Schizophrenia, bipolar, and depression	Lack of engagement in post-discharge aftercare services was a key predictor for readmission.
Hodgson et al., 2001	UK	Retrospective chart review	3404	16–64 years	Psychiatric disorders	Predictors for readmission were being unmarried and having a psychosis diagnosis. The readmission rate was 49% for patients with affective disorders, 42% for schizophrenia, and 39% for personality disorders.
Hung et al., 2017	Taiwan	Retrospective chart review study	138	38.73 years	Schizophrenia and schizoaffective disorder	The one-year and 3-months readmission rates were 33.3% and 15.2%, respectively. Predictors were an unmarried status, previous history of involuntary admission, longer involuntary admission days, and shorter total admission days.
Joanna et al., 2016	USA	Retrospective cohort analysis	1450	18–64 years	Schizophrenia	The rate of readmission within the 60-day post-discharge period was 25%. Long-acting injections (LAIs) were found to significantly reduce the risk of readmission by 16% compared to oral antipsychotics.
John et al., 1997	USA	Observational study	255	30.8 years	Psychiatric conditions	Predictors for readmission were patients with more significant impairment in self-care, more severe symptoms, and more persistent illnesses.
Joyce et al., 2019	USA	Retrospective cross-sectional study	783	12–17 years	Psychiatric conditions	The readmission rate was 26% at two years post-discharge, 22% at one year, 18% at 180 days, 12% at 90 days, and 6% at 30 days. Predicting factors were a diagnosis of PTSD, severe lifetime SI, and lack of treatment alliance.
Lee et al., 2017	Malaysia	Observational study	149	37.8 years	Psychotic disorder, bipolar disorder, major depression disorder, mental sub-normality and substance abuse	Patients were readmitted within three months of their last discharge. Predictors were male, single, unemployed, taken care of by family, and achieved secondary education level with a mean age of 37.89 years.
Lewis et al., 2019	Australia	Comparative study	2833	42.08 years	Personality disorder	Readmission was within 28 days after discharge. Predictors were a personality disorder diagnosis and psychotic disorders.
Lorine et al., 2015	USA	Retrospective chart review	207	18–65+ years	Schizophrenia, MDD, seasonal affective disorder (SAD), and psychosis	Risk factors for readmission within 15 days were diagnosis of schizophrenia and or seasonal affective disorder (SAD).
Maali et al., 2018	Australia	Case study	62,255	16–75 years	Psychiatric and medical diagnosis	Predictors for readmission were the previous history of healthcare utilization, the urgency of the index admission, old age, comorbidities related to cancer, psychosis, drug abuse, abnormal pathology results at discharge, and being unmarried and a public patient.
Manu et al., 2014	USA	Cohort	945	41.5 years	Schizophrenia spectrum disorders, bipolar disorder, and depressive disorder.	The one year readmission rate was 24%. Higher BMI, diagnosis of schizophrenia and schizoaffective disorder, shorter length of stay, and treatment with clozapine were key predictors of readmission.
Maples et al., 2012	USA	Observational study	345	36.3 years	Schizophrenia, bipolar, and depression	The use of hospital and crisis or emergency services reduced significantly within the 30 days of discharge.
Marcus et al., 2017	USA	Retrospective longitudinal cohort analyses	71,776	18–64 years	Schizophrenia and bipolar disorder	Outpatient visits 30 days after discharge were associated with a lower hospital readmission risk during the following 90 days. Assertive hospital discharge planning to secure outpatient visits after hospital discharge is needed for these patient populations.
Mark et al., 2013	USA	Retrospective cross-sectional study	121,275	18–65 years	Substance use disorder	The annual readmission rate was 11%. The hospital-level factors that reduced readmission rates were patients with follow-up at a community mental health center (CMHC), and longer lengths of stay. Prior to the inpatient stay, psychotic illness, substance use disorders, and medical comorbidities were predicted.
Mellesdal et al., 2010	Norway	Prospective cohort study	1245	41.6	Psychiatric diagnosis	Fifty-four percent (54%) of the index admissions and 62% of the readmissions were related to suicide risk. Other predictors were substance use disorders, personality disorders, prior psychiatric hospitalization, unemployment, and receipt of social benefits.
Mgutshini et al., 2010	USA	An explorative study	59	18–74 years	Psychiatric diagnosis	The predictor of readmission was non-adherence with prescribed medication.
Miller et al., 2020	Canada	Retrospective cross-section study	3825	3–19 years	All psychiatric conditions	The rate of readmission within 90 days following discharge was 57.3%. Predictors were older age, being male, higher socioeconomic status (SES), referral to care by a medical practitioner, discharge to another health facility, psychosis, and previous psychiatric admission.
Moos et al., 1994	USA	Cross-sectional Study	85	56	Substance abuse	Predictors of readmission were being married, diagnoses of alcoholic psychosis and treatment in the index episode, and discharge against medical advice (AMA).
Moos et al., 1998	USA	Retrospective chart review and file analysis	16,066	61.3 years	Substance abuse	Four-year readmission rates ranged from 57% to 70%. Predictors for readmission were younger age, unmarried status, more prior service use, alcohol psychosis or psychiatric diagnoses, treatment in a psychiatric unit, and shorter hospital stay.
Munley et al., 1978	USA	Retrospective cohort study	202	39.8 years	Substance abuse	Predictors for readmission were the number of prior psychiatric hospitalizations and diagnosis of depression upon admission.
Nagata et al., 2019	Japan	Retrospective chart review	526	48 years	Substance abuse	Following discharge, the cumulative admission rate to local psychiatric hospitals was 21.8% after six months and 37.6% after one year. Predicting factor was patients who had been discharged from their Medical Treatment and Supervision Act [MTSA] order but transferred to a general psychiatric hospital.
Nahid et al., 2020	Qatar	Retrospective chart review	380	1–64 years	All psychiatric conditions	Eleven percent (11%) were readmitted within 30 days of discharge. The predictors were single, male and unemployed, and poor or no compliance to medication after discharge.
Olfson et al., 1999	USA	Retrospective cross-sectional study	316	18–64 years	Schizophrenia and schizoaffective disorder	The rate of readmission was 24.4% within three months of hospital discharge. Predictors were male subjects, persons who had never married, and individuals with relatively low levels of formal education.
Ortiz et al., 2019	USA	Retrospective chart review and file analysis	60,254	38 years	Psychiatric diagnosis	Eight percent (8%) of discharges were readmitted to the same hospital within 30 days of discharge. Factors predicting readmission were white, non-Hispanic, not married, voluntarily admitted, with the length of stay, LOS 8–31 days, LOS 32–92 days, and schizophrenia or other psychotic disorders or personality disorder.
Osborn et al., 2021	England	Cohort study	231,998	16–54 years	Severe psychosis	The rate of readmission was 21.4% within six months. Predictors were female, older, single, from black or mixed ethnic groups, or more deprived areas. Clinical predictors included shorter index admission, psychosis and being an inpatient at baseline.
Owen et al., 1997	Australia	Observational study	135	35.2 years	Schizophrenia, schizoaffective disorder, bipolar, and depression	In the follow-up period, 5% of patients had been admitted to a drug and alcohol admission unit, and 3% had gone to jail. There was no relationship between patient demography, diagnosis, level of symptoms or functioning at discharge, patient attitude to follow-up or patient “likeability” and rehospitalization prediction.
Pablo et al., 1986	Canada	Retrospective chart review and file analysis	150	17–65 years	Schizophrenia, affective disorders	Only 3% of the patients had any readmissions after their 1981 readmission. The average number of readmissions per patient was only 0.63.
Patricia et al., 2002	USA	Randomized control trial	80	39.2	Psychosis	The readmission rate was reduced by 50% over a 4-month period in patients with positive symptoms of psychosis.
Payne et al., 2009	Scotland	Retrospective cross-sectional study	50,891	15–65 years	Self-poisoning	The 1-year readmission rate was 12.2%. Predictor factors were previous psychiatric hospital treatment and diagnosis of personality disorder.
Phillips et al., 2020	USA	Retrospective cohort study	6797	6–17 years	Mood disorders	Publicly insured, mood disordered, and psychiatrically hospitalized youth were predictors for readmissions within six months of the initial hospital discharge with a readmission rate of 13.8%.
Pieterse et al., 2016	South Africa	Retrospective cohort study	99	12–19 years	Schizophrenia, spectrum bipolar type 1, and substance-induced psychotic disorder	Thirty-six percent (36%) were readmitted during the study period. The predictor factor was having a prior admission. No association was seen between the type of diagnosis and readmission.
Popiolek et al., 2018	Denmark	Population based study	1255	52.2	Bipolar disorder	The rates for readmission were 29%, 41%, and 52% at 3, 6, and 12 months, respectively, post-discharge. Predictors were a history of multiple psychiatric admissions, lower age, and post-discharge treatment with antipsychotics or benzodiazepines.
Puntis et al., 2016	UK	Retrospective chart review	323	18–65 years	Psychosis	Patients who had a higher proportion of clinical correspondence copied to them spent fewer days in the hospital.
Regis et al., 2016	Brazil	Survey	6261	1–60 years	Psychiatric patients	Within 90 days after discharge, the readmission rate ranged from 16.1 to 20.9%. The predictors for early readmission included the diagnosis of depressive, bipolar, psychotic, and non-alcohol-related disorders, younger ages, and unemployment.
Roaldset et al., 2014	Norway	Prospective observational study	196	38–46 years	Psychiatric diagnosis	Patients exhibiting three or more deliberate self-harm (DSH) related rehospitalizations (repeated DSH, DSH-R) constituted 24% of the total sample of DSH patients and 9% of all patients but accounted for 83% of DSH-related rehospitalizations and 44% of all rehospitalizations.
Romansky et al., 2003	USA	Randomized case study	500	3 to 21 years	Childhood severity of psychiatric illness (CSPI)	Twenty-one percent of the girls and 22% of the boys were readmitted. Learning disability and mental retardation were crucial predictors for readmission.
Romer et al., 2018	Denmark	Record-linkage study	634	34.6 years	Schizophrenia, psychosis, and substance use disorder	Of all patients, 78.7% were readmitted for schizophrenia within 12 months of discharge. Predictors were the use of amphetamine and cannabis, a history of schizophrenia and drug use disorder (DUD).
Rylander et al., 2016	USA	Retrospective chart review	693	38 years	Psychiatric diagnosis	Predictors for readmission were male gender with suicidal ideation on admission, a diagnosis of a psychotic disorder with a prior medical admission, and suicidal ideation with a comorbid personality disorder.
Schmutte et al., 2010	USA	Matched-control survival analysis	150	42.2 years	Psychiatric diagnosis	Thirty percent (30%) were rehospitalized during the 12-month observation period. Predictors were unemployment and living in supervised residential facilities.
Shadmi et al., 2018	Israel	Cohort study	2842	46.9years	Schizophrenia	Quality of life (QoL) was a significant predictor for future hospitalization within six months, and self-report of the impact of symptoms on functioning significantly predicted 12-month hospitalization.
Shaffer et al., 2015	USA	Retrospective comparative study	149	38.8 years	Anxiety disorder, bipolar disorder, schizophrenia, and depression	Longer-term readmission rates were lower in the brief critical time intervention (BCTI) cohort but were not significantly different from the comparison cohort (44% versus 52%). Brief critical time intervention (BCTI) decreased early psychiatric hospital readmission rates for individuals at high risk of readmission.
Silva et al., 2009	Brazil	Case-control study	307	18–69 years	Psychiatric conditions	Individuals referred to community psychosocial support units after their most recent discharge had about a 20% lower risk of multiple readmissions than those referred to usual outpatient care. Predictors of readmission were diagnosis of schizophrenia or psychotic symptoms, younger age at first admission and a more significant number of previous admissions.
Silva et al., 2020	Portugal	Retrospective cross-sectional study	3872	65+ years	Psychiatric diagnosis	Retired, psychotic, and patients with compulsory admission are predicted to have more than one admission within a year. Older age and having a secondary or higher education reduced the risk of admissions.
Smith et al., 2015	USA	Quasi-experimental	164,544	17–65 years	Mental health and substance abuse conditions	The readmission rate for mental health and substance abuse (MHSA) diagnoses was 16.4% within the 12 months after discharge. Predictors were alcohol or drug dependence, dementia, psychotic disorders, autism, impulse control disorders, and personality disorders.
Soni et al., 1994	UK	Case study	88	47.6 years	Chronic schizophrenia	Predictors for readmission were early age of onset of illness, the severity of positive and affective symptoms, current neuroleptic dose and total Involuntary Movement Scale (AIMS) score.
Soyka et al., 2013	Germany	Retrospective cross-sectional study	5,300,000	49 years	Alcohol-induced psychotic disorder	The readmission rate for four years is 62.4%. The key predictors were chronic schizophrenia and mental or behavioral disorders.
Tedeschi et al., 2019	Italy	Comparative study	63,419	18–65 years	Psychiatric diagnosis	The 1-year readmission rate was 43.0%. Predictors for readmission were: admission in the same local health districts (LHDs) as a residence, psychotic disorder, greater length of stay (LoS), and higher rate of public beds in the LHD.
Thomas et al., 2018	USA	Retrospective observational study	240	18–65 years	Schizophrenia, schizoaffective disorder, or bipolar disorder	The rate of readmissions for those who received the LAI and those who did not were 43.1% and 56.95, respectively. Patients who received the LAI at a frequency of one month or longer had a significant longer survival time without readmission compared to those with a shorter administration frequency (mean 307.9 and 245.0 days).
Trask et al., 2016	USA	Retrospective chart study	569	6–18 years	Childhood psychiatric disorders	Seventy percent (70%) of youths with a psychiatric hospitalization received aftercare and 28% were rehospitalized within six months of discharge. Predictors were having a diagnosis of schizophrenia and receiving more days of day treatment reduced the risk of rehospitalization.
van Alphen et al., 2017	USA	Retrospective observational study	165	13–19 years	Depression	Overall, 12.1% of patients were rehospitalized within the period. The predictor was frequent self-injurious behaviors in the month prior to hospitalization.
Volpe et al., 2018	Brazil	Retrospective cross-section study	19,723	37.4 years	All psychiatric conditions	The rates for readmissions were 5.7% for less than seven days, 5.9% for 8–30 days, and 18.9% for 31–365 days. Young male patients and those residing outside the capital were predictors for readmission. Psychotic disorders, mood disorders, and neurotic disorders were protective factors for readmission.
Xu et al., 2018	USA		973,078	52–70 years	Epilepsy	Less than one percent were readmitted. Predictors were psychiatric comorbidities, epilepsy, and risk of suicide.
Yedlapati et al., 2018	USA	Cross-sectional and retrospective analyses	163,143	50.4 years	Alcohol withdrawal	The yearly readmission rate was 60%. Predictors for 30-day readmission and multiple readmissions were discharged against medical advice (AMA), comorbid psychosis and low socioeconomic status.
Yussuf et al., 2008	Nigeria	Retrospective record study	502	21–40 years	Psychiatric patients	Within the study period, 41.4% were readmitted. Predictors for readmission were a young age, a longer length of stay, multiple admissions, and a diagnosis of schizophrenia.
Zhang et al., 2011	Australia	Retrospective study	249	39 years	Psychotic illness	Predictors for readmission were a history of previous frequent admissions, a risk to others at the index admission and alcohol intoxication. More active and assertive treatment in the community post-discharge decreased the risk of readmission.
